# Retinal Cyclic Nucleotide-Gated Channels: From Pathophysiology to Therapy

**DOI:** 10.3390/ijms19030749

**Published:** 2018-03-07

**Authors:** Stylianos Michalakis, Elvir Becirovic, Martin Biel

**Affiliations:** Center for Integrated Protein Science Munich (CIPSM), Department of Pharmacy—Center for Drug Research, Ludwig-Maximilians-Universität München, Butenandtstr, 5-13, 81377 Munich, Germany; elvir.becirovic@cup.uni-muenchen.de (E.B.); mbiel@cup.uni-muenchen.de (M.B.)

**Keywords:** CNG, cyclic nucleotide-gated channel, vision, channelopathies, Ca^2+^, knockout, photoreceptor, cGMP, gene therapy

## Abstract

The first step in vision is the absorption of photons by the photopigments in cone and rod photoreceptors. After initial amplification within the phototransduction cascade the signal is translated into an electrical signal by the action of cyclic nucleotide-gated (CNG) channels. CNG channels are ligand-gated ion channels that are activated by the binding of cyclic guanosine monophosphate (cGMP) or cyclic adenosine monophosphate (cAMP). Retinal CNG channels transduce changes in intracellular concentrations of cGMP into changes of the membrane potential and the Ca^2+^ concentration. Structurally, the CNG channels belong to the superfamily of pore-loop cation channels and share a common gross structure with hyperpolarization-activated cyclic nucleotide-gated (HCN) channels and voltage-gated potassium channels (KCN). In this review, we provide an overview on the molecular properties of CNG channels and describe their physiological role in the phototransduction pathways. We also discuss insights into the pathophysiological role of CNG channel proteins that have emerged from the analysis of CNG channel-deficient animal models and human CNG channelopathies. Finally, we summarize recent gene therapy activities and provide an outlook for future clinical application.

## 1. Introduction

**Figure 1:**
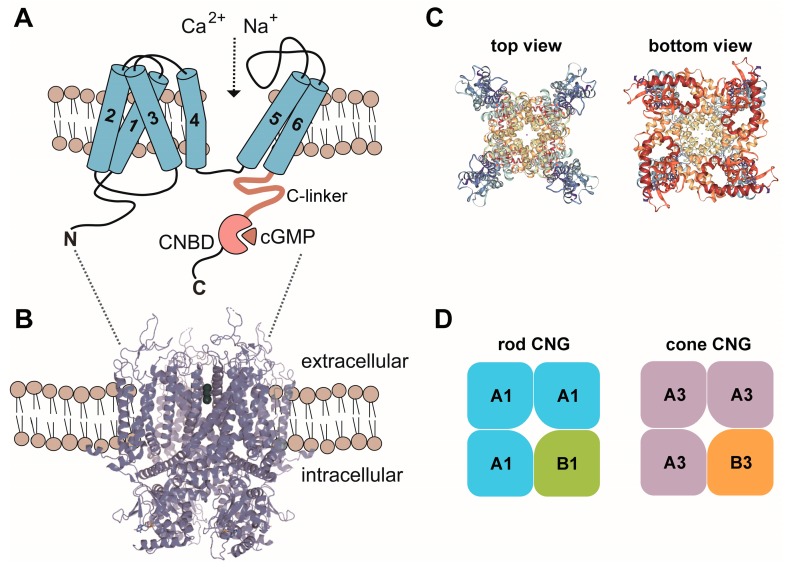
(**A**) Membrane topology of CNG channel subunits. 1–6, transmembrane segment 1–6; C, carboxy-terminus; CNBD, cyclic nucleotide binding domain; N, amino-terminus. (**B**) Model of the CNG channel complex embedded in the plasma membrane based on the TAX-4 structure. (**C**) Top and bottom views of the tetrameric TAX-4 *C. elegans* CNG channel complex. (**D**) Subunit composition of the CNG channels from rods and cones. A1, CNGA1; A3, CNGA3; B1, CNGB1; B3, CNGB3. Structures in this figure were generated with the RSCB PDB 3D View tool (www.rcsb.org/3d-view/) based on PDB 5H3O.

Cyclic nucleotides, such as cAMP and cGMP, regulate the activity of several classes of proteins including cyclic nucleotide-binding domain (CNBD)-containing cation channels. Structurally, cyclic nucleotide-regulated channels belong to the superfamily of pore-loop cation channels [1]. In vertebrates, three subfamilies of CNBD-containing ion channels exist: the CNG, the HCN and the KCN channels [2,3]. These channels share a similar principal architecture but differ from each other in terms of their activation mode and ion selectivity. Cyclic nucleotide-gated (CNG) channels are strictly ligand-gated because their opening requires the binding of cGMP or cAMP. CNG channels have been also found in several non-vertebrate species including insect species and *C. elegans*. In mammals, the CNG channel family comprises six homologous members (*CNGA1-4*, *CNGB1* and *CNGB3*) encoding for structurally similar A and B subunits that assemble in distinct combinations into cell type-specific heterotetrameric complexes [4] (Figure 1). The A subunits confer the principal channel properties, whereas the B subunits are essential for proper outer segment localization and contribute specific biophysical properties (e.g., fast gating kinetics) to the native channel complex [2].

## 2. Structure, Basic Properties and Activation of CNG Channels

CNG channels form a distinct branch within the superfamily of voltage-gated-like (VGL) channels [1,5]. In mammals, the CNG channel family comprises six homologous members, which are classified as A subunits (CNGA1-4) and B subunits (CNGB1 and CNGB3). A subunits and B subunits share the same principal membrane topology. Each of these subunits possesses a transmembrane channel core consisting of six α-helical segments (S1–S6), a reentrant pore (P) loop between S5 and S6, and cytosolic N- and C-termini. The CNBD is located in the C-terminus and is connected to the S6 by the C-linker. Native CNG channels in photoreceptor outer segments are heterotetramers assembled by three A subunits (CNGA1 in rods and CNGA3 in cones) and one B subunit (CNGB1 in rods and CNGB3 in cones) [6,7,8,9]. So far, no structure of mammalian CNG channels is available. However, a structure of the CNG channel from *C. elegans* TAX-4 has been recently determined by single-particle electron cryo-microscopy [10]. Like other CNBD-containing channels [3], CNG channels are tetramers with the four subunits arranged around the centrally located pore [10]. The ion conducting pore is lined by the P-loops and the S6 segments of the four subunits. The TAX-4 voltage-sensor-like (VSL) domain is unique among known VSL structures as it appears segmented. This segmentation might preclude voltage-dependent movements within the membrane and thus help explain the lack of voltage-dependent gating of CNG channels [10]. However, the TAX-4 structure only reflects the cGMP-bound open state and a closed state structure is missing. Based on comparisons of available open and closed state structures from related channels and previous mutagenesis studies, activation of CNG channels is thought to involve coordinated movements of at least three basic elements, the CNBD, the C-linker and the channel gate [10,11,12,13,14,15]. Binding of cyclic nucleotides to the CNBD leads to a rotational change of the entire C-terminus relative to the pore. The C-linker, a domain that allosterically couples cyclic nucleotide-binding to the channel gate also follows this rotation and partially moves upwards. The channel gate located in the intracellular portion of the S6 segment is constricted and maintained in the closed state presumably by steady forces from the C-linker [16,17]. Upon binding of the ligand, the movements described above relieve the inhibitory forces of the C-linker and the channel pore becomes delated, thereby allowing for ion permeation.

While all six mammalian CNG channel subunits share significant sequence homology, only CNGA1, CNGA2 and CNGA3 can form functional homotetrameric channels in heterologous expression systems. The remaining subunits (CNGA4, CNGB1 and CNGB3) do not assemble to functional homotetramers. However, when present in heterotetrameric CNG channel complexes, they confer key functional properties specific for native CNG channels (e.g., single channel flickering, altered affinity for cyclic nucleotides, distinct permeation properties, regulation by Ca^2+^) (for detailed review see [2,18]). 

CNG channels are non-selective for several monovalent or divalent cations, such as Na^+^ and K^+^, Ca^2+^ and Mg^2+^. However, Ca^2+^ and Mg^2+^ can also act as a voltage-dependent blocker of monovalent cation permeability [19,20]. Another characteristic feature of CNG channels is the lack of desensitization or inactivation upon exposure to cyclic nucleotides. In addition, CNG channels show Ca^2+^-dependent feedback inhibition, which is thought to be calmodulin (CaM)-mediated [21,22]. In rods, Ca^2+^-inhibition is conferred by binding of CaM to an IQ-type binding site located at the N-terminus of the CNGB1 subunit [23,24]. In cone photoreceptors this inhibitory effect is more pronounced and most likely mediated by another Ca^2+^-binding protein termed CNG-modulin [25].

## 3. Signal Transduction in Photoreceptors

**Figure 2:**
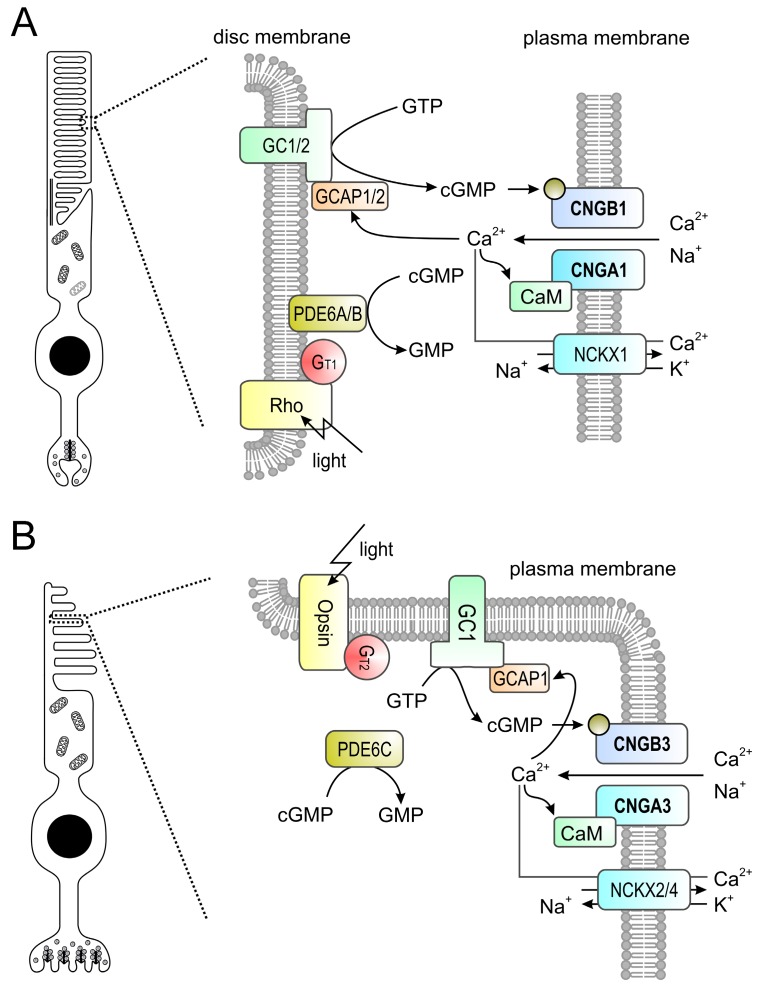
(**A**,**B**) Phototransduction in mouse rod (**A**) and cone (**B**) outer segments. The principle of the phototransduction is similar in both cell types. In the dark, the cyclic nucleotide-gated (CNG) channel (CNGA1/B1 in rods and CNGA3/B3 in cones) of the outer membrane is kept open by high concentrations of cyclic guanosine monophosphate (cGMP) produced by retinal guanylyl cyclases (GCs) 1 and 2 (GC1/2 in rods, GC1 only in cones) present in the disc membrane. The resulting influx of Na^+^ and Ca^2+^ depolarizes the plasma membrane. Light activates rhodopsin (Rh) which in turn activates transducin (G_t1_ in rods, G_t2_ in cones) whose alpha subunit activates a phosphodiesterase (PDE6A/B in rods, PDE6C in cones) leading to hydrolysis of cGMP. The drop in the cGMP concentration leads to the closure of the CNG channel yielding to membrane hyperpolarization. Ca^2+^ is an important regulator of phototransduction. At high concentrations Ca^2+^ binds to guanylyl cyclase-activating proteins (GCAPs; GCAP1/2 in rods, GCAP1 only in cones) leading to an inhibition of guanylyl cyclases. High levels of Ca^2+^ also lead to a slight reduction of the cGMP affinity of the CNG channel via CaM-mediated feedback inhibition. Ca^2+^ is cleared from the outer segment via a Na^+^-Ca^2+^-K^+^-exchanger (NCKX1 in rods, NCKX2/4 in cones). At low Ca^2+^ levels GCAPs switch to the Ca^2+^-free form that is an activator of GCs.

Rod and cone photoreceptors possess similar, but distinct phototransduction pathways which fulfill the required initial processing of visual stimuli at different ambient light conditions (Figure 2). Rods mediate vision at dim light levels, whereas vision at higher light levels (e.g., daylight) is provided by cones with minor contribution from rods. The cone visual system also enables color vision as it can discriminate between wavelengths by comparing inputs from two (in most vertebrates) or three (in humans and some non-human primates) types of cones equipped with different cone opsin variants (short-, medium- and long-wave sensitive) with distinct spectral sensitivities [26,27]. In both rods and cones, signal transduction follows the same principle and is facilitated by enzymes controlling the levels of the second messenger cGMP. cGMP in turn controls the activation of the CNG channel present in the plasma membrane of the photoreceptor outer segment (Figure 1). In the dark, constant activity of transmembrane guanylyl cyclases [28,29] results in high levels of cGMP, which maintains the CNG channel in an opened conformation. As mentioned in the previous section, CNG channels carry a steady non-inactivating Na^+^ and Ca^2+^ current (“dark current”) which depolarizes the photoreceptor promoting glutamate release at the photoreceptor synaptic terminal. Following light stimulation photopigments (opsins) initiate a G protein (transducin) mediated signaling cascade leading to activation of a cGMP phosphodiesterase (PDE6). PDE6 hydrolyses cGMP and thereby leads to closure of the CNG channel. Consequently, the photoreceptor hyperpolarizes and the synaptic glutamate release decreases. CNG channels provide the only source for Ca^2+^ influx into rod and cone outer segments [2,30,31]. Ca^2+^ entry is balanced by Ca^2+^ extrusion through the activity of a Na^+^/Ca^2+^-K^+^ exchanger [31,32,33,34,35]. Thus, light stimulation of photoreceptors results in a decrease of the intracellular Ca^2+^ concentration because it shuts down Ca^2+^ entry via the CNG channels while the Na^+^/Ca^2+^-K^+^ exchanger continues to clear Ca^2+^ from the cytosol. The reduced Ca^2+^ level upon light absorption contributes to the recovery from light response by several mechanisms including phosphorylation of visual pigments and PDE6, as well as restoration of cGMP levels by guanylyl cyclase activating proteins (GCAPs; [36])-mediated activation of the guanylyl cyclase. 

Photoresponses measured from isolated rods and cones differ in sensitivity and kinetics [2,37]. Rod and cone CNG channels share the same principle functional properties and differ only in some specific features. Notable examples are the higher Ca^2+^-permeability of the cone CNG channel and the stronger Ca^2+^-dependent inhibition of the ligand-sensitivity in the rod CNG channel [2,38]. However, these differences cannot explain the differences in rod and cone photoresponses, which most likely result from multiple distinct signaling cascade molecules and distinct morphological characteristics (e.g., outer segment compartmentalization).

## 4. In Vivo Analysis of CNG Channel Function: Analysis of Genetic Mouse Models and Human Channelopathies

Genetic studies have dramatically increased our knowledge on the significance of retinal CNG channels. The major findings of these studies will be summarized in the following sections. 

### 4.1. CNGA1 and CNGB1

Mutations in the rod-specific *CNGA1* or *CNGB1* genes cause autosomal recessive retinitis pigmentosa (RP) [39,40] (Table 1). RP comprises a genetically diverse group of progressive degenerative retinal diseases affecting the photoreceptors of the retina [41,42]. The most common symptoms of RP include night blindness, progressive concentric reduction of the visual field, and abnormal accumulation of pigmentation in the retina [43]. Fast progressing RP can lead to legal blindness in advanced stages. So far, RP has been mapped to >60 genes (http://www.sph.uth.tmc.edu/Retnet, 22 February 2018) encoding proteins involved in the visual transduction pathway or required for the maintenance of the rod architecture. Depending on the genetic background, mutations in *CNGA1* account for 1–8% of cases of autosomal recessive RP (arRP) [40,41,44,45,46]. Most of the identified *CNGA1* mutations cause deletions of important functional domains or result in defective membrane trafficking [2,40,47,48]. As a result, the affected patients lack rod-mediated light responses. Mutations in *CNGB1* account for approx. 4% of arRP cases [39,41,49,50]. Although the known *CNGB1* mutations cause only minor deletions or single amino acid substitutions, the phenotype is comparable to the severe RP phenotype in *CNGA1* patients. So far, only a few *CNGB1* mutations have been functionally characterized. These mutations lead to protein instability, impaired channel targeting or functionally inactive CNG channels [39,49,51,52].

Mice lacking *Cnga1* have not been described so far. However, transgenic mice overexpressing *Cnga1* antisense mRNA were generated [53]. Although no functional characterization of these mice has been reported, histological analysis revealed some morphological features of RP (e.g., reduced number of photoreceptors, apoptotic death of retinal cells). A naturally occurring *Cnga1* mutation has been recently identified in a Shetland sheepdog breed with progressive retinal atrophy [54]. However, functional characterization of the retinal phenotype is still missing. 

Genetic deletion of *Cngb1* in mice results in a phenotype that recapitulates the principal pathology of RP patients. In particular, *Cngb1* knockout (KO) mice have strongly diminished rod photoreceptor function and impaired rod-mediated vision [55,56,57]. The early functional defects are followed by a progressive degeneration of rods and secondary degeneration of primarily non-affected cones. The degeneration process is rather slow and results in loss of about 50% of rods at 6 months and 90% at 12 months of age [55,57,58]. At the cellular level, the loss of *Cngb1* induces down-regulation of several proteins of the phototransduction cascade and degradation of the CNGA1 subunit. A naturally occurring *Cngb1* mutation was also identified in a Papillon dog breed with markedly reduced or absent rod function, and accompanied by progressive retinal degeneration [59]. Comparative analysis suggested that *CNGB1*-deficient RP patients and mouse and dog models have a similar phenotype characterized by early loss of rod function and slow rod photoreceptor degeneration along with a secondary decline in cone function [58].

### 4.2. CNGA3 and CNGB3

Mutations in either *CNGA3* [60] or *CNGB3* [61,62] cause achromatopsia (ACHM), also known as rod monochromatism or total color blindness (Table 1). ACHM is an inherited eye disease associated with congenital absence of cone photoreceptor function. In contrast to color blindness, in which changes in expression of opsin genes merely affect spectral sensitivity but not the physiology of photoreceptors [63,64], the complete unresponsiveness of cones in ACHM has grave consequences for vision, particularly with respect to the densely cone-packed human fovea. Affected individuals suffer from strongly impaired daylight vision, photophobia, nystagmus, and lack of color discrimination [65,66,67]. The prevalence of ACHM is approximately 1:30,000 [68,69,70]. Currently, six genes have been linked to ACHM: *ATF6, CNGA3, CNGB3, GNAT2, PDE6C* and *PDE6H*. Up to 80% of the patients carry mutations in the genes *CNGA3* and *CNGB3* encoding the two subunits of the cone CNG channel. To date, more than 100 mutations in *CNGA3* and more than 50 mutations in *CNGB3* were found to cause inherited ACHM in humans [71]. Mutations in *CNGB3* (ACHM1) are more common in the general western population and account for almost 50% of ACHM cases [72]. Most *CNGB3* mutations are nonsense, frameshift or splice mutations resulting in truncated or strongly impaired channel proteins [68,69]. A missense mutation in the *CNGB3* gene (S435F) was identified in colorblind individuals originating from the Pingelap atoll of Micronesia [62]. In this small island ACHM is very frequent and affects nearly 10% of the native population [73]. Approximately 28% of patients in the western population carry mutations in *CNGA3* (ACHM2). In Middle East and Arabic populations, mutations in *CNGA3* account for up to 60% of ACHM cases. The majority of *CNGA3* mutations are missense mutations affecting only single amino acid residues of the protein [68,69]. 

Loss of function mutations in *CNGA3* result in non-functional cone CNG channels because CNGB3 cannot form functional CNG channels in the absence of CNGA3 [2,47]. Genetic inactivation of *Cnga3* in mice leads to selective loss of cone-mediated light responses [74] accompanied by progressive degeneration and cell death of cones [75]. An early hallmark of cone degeneration is the strong accumulation of the second messenger cGMP, suggesting its involvement in the process of degeneration [76,77]. Cone degeneration affects M- and S-cones differentially and cell death proceeds significantly faster in ventral and nasal (S-cone-rich) than in dorsal and temporal (M-cone-rich) parts of the retina. Ventral cones are almost completely missing after the third postnatal month whereas residual dorsal cones are present even in aged knockout mice [75]. In addition, a naturally occurring mouse model of achromatopsia—the *cpfl5* mouse with a *Cnga3* point mutation—was described with a phenotype similar to the *Cnga3* KO mouse [78]. Moreover, a sheep model of ACHM2 was identified with diminished cone, but normal rod function [79,80]. Affected lambs were homozygous for a *Cnga3* mutation which results in a premature stop codon [79]. Recently, two spontaneous canine models of ACHM2 have been described [81]: a German shepherd, carrying a *CNGA3*/p.R424W mutation, and a Labrador retriever with a *CNGA3*/p.V644del mutation.

Various animal models also exist for *CNGB3*-linked ACHM. Knockout of *Cngb3* in mice strongly impairs cone function and leads to progressive cone degeneration [82,83]. A residual cone function can be observed in this model, most likely conferred by irregular homomeric CNGA3 channels. In addition to the *Cngb3* knockout (KO) mouse, multiple naturally occurring canine models exist that carry recessive mutations in exon 6 or a genomic deletion of the entire *Cngb3* gene [84]. The latter results in a disease sharing the same clinical phenotype as human patients characterized by day blindness and absence of cone function [85,86,87,88].

## 5. Gene Therapy for the Treatment of CNG Channelopathies

No curative treatment for CNG channelopathies (ACHM or RP) exists thus far and clinical management is currently limited to specialized genetic counseling, the use of low vision aids, tinted contact lenses or glasses to reduce symptoms of photophobia [70,107]. Our improved knowledge on CNG channel biology and the availability of suitable animal models together with the advent of recombinant adeno-associated virus (rAAV) vectors as efficient and safe retinal gene delivery tools has led to the initiation of several promising gene therapy programs (Table 1). 

ACHM and RP caused by mutations in CNG channels are both inherited in an autosomal recessive manner. Therefore, most treatment approaches for CNG channelopathies focus on so-called gene supplementation. Gene supplementation aims at complementing affected photoreceptor cells with a healthy copy of the disease-causing gene. In the following paragraphs, we will provide an overview on rAAV vectors, review current preclinical activities and finally summarize ongoing clinical trials.

rAAV vectors are based on AAVs, which are small (25 nm diameter), non-enveloped and non-pathogenic parvoviruses with a genome packaging capacity of approx. 4.7 kb [108]. rAAV vectors are devoid of most AAV genomic sequences and contain only short palindromic sequences, so called inverted terminal repeats (ITRs), which flank the therapeutic gene expression cassette [108]. In recent years, rAAV vectors have evolved as the gold standard gene delivery vector for retinal photoreceptors. Their success is based on the fact that they are easy to produce at large scale and their small genome can be easily manipulated [109,110]. Moreover, a large and growing number of naturally occurring or engineered AAV capsids are available allowing for packaging of pseudotyped rAAV of different serotypes with distinct properties, e.g., specificity for different cell types [111,112]. Finally, rAAVs are generally considered as very safe gene therapy vectors with low immunogenicity and toxicity [113,114]. After transduction, in contrast to the native unmodified AAV that integrates into a preferred genome site, the rAAV vector genome stays predominantly in an episomal state and no considerable integration into the host genome has been reported [115,116,117].

## 6. Gene Therapy for CNG Channel-Linked RP

So far, no gene therapy approaches have been reported for *CNGA1*-linked RP (RP49). This is most likely due to the lack of well-characterized animal models for this form of RP. For *CNGB1*-linked RP (RP45) successful proof-of-concept studies for rAAV-based gene supplementation have been reported in both the *Cngb1* KO mouse model [90] and the *Cngb1* mutant dog model [58]. To enable efficient packaging and rod-specific expression of the relatively large full-length *Cngb1* cDNA (~4 kb), the two studies used an rAAV expression cassette with a short rod- [90] or photoreceptor-specific [58] promoter combined with short regulatory elements (e.g., SV40 polyA). In both species, subretinal injection of therapeutic rAAV gene supplementation vectors (serotype 5 or 8) led to (i) restoration of CNG channel expression and localization, (ii) gain of retinal function and preservation of retinal morphology and (iii) improvement of vision-guided behavior. Apart from the expression of full-length CNGB1, rAAV treatment also restored normal levels of the otherwise degraded endogenous CNGA1 subunit of the rod CNG channel in this mouse model. Both proteins co-localized in rod outer segments and formed regular CNG channel complexes within the treated area of the *Cngb1* KO or mutant retina, leading to significant morphological preservation and a delay of retinal degeneration. In the electroretinographic analysis, substantial restoration of rod-driven light responses was observed. Finally, treated *Cngb1* KO mice as well as *Cngb1* mutant dogs performed significantly better than untreated controls in rod-dependent vision-guided behavior tests [58,90]. These promising results facilitated the initiation of translational studies, which might culminate in a first in man clinical trial.

## 7. Gene Therapy for CNG Channel-Linked ACHM

Successful preclinical proof-of-concept animal studies for rAAV-based gene supplementation therapy exist for *CNGA3* and *CNGB3* [76,78,92,93,101,102,103]. In these studies wildtype or tyrosine mutant AAV2, AAV5 or AAV8 vectors expressing the corresponding wildtype cDNAs under control of cone-specific or ubiquitous promoters were used to evaluate rAAV-mediated gene supplementation as a potential treatment of ACHM. The studies utilized engineered or naturally occurring mouse, dog or sheep models of ACHM and reported beneficial effects of the treatment on cone-specific visual function. Treated animals showed cone-driven light responses and showed structural preservation of cone photoreceptors. Our group studied in detail the effect of rAAV-mediated gene supplementation in *Cnga3* KO mice [76,93]. In addition to the gain of retinal function, we could show that the treatment also had beneficial effects on retinal morphology and degenerative cellular processes. For instance, the therapy normalized pathologically elevated cellular cGMP levels in cones, delayed cone cell death and diminished the inflammatory response of Müller glia cells that is a hallmark of retinal degenerations [75]. Follow-up studies suggested that the therapeutic effect was long-lasting and could be applied at more advanced disease stages (e.g., at three months of age) [93]. 

rAAV-based gene supplementation has proven equally effective in preclinical models of both *CNGA3*- [76,78,92] and *CNGB3*-linked ACHM [101,103] with the only exception being the reduced treatment efficiency of the treatment in advanced stage *Cngb3* mutant dogs [101]. However, the efficiency in this dog model could be improved when rAAV gene supplementation was combined with the administration of ciliary neurotrophic factor (CNTF) which is known to cause a temporal deconstruction of photoreceptor outer segments [102]. 

The very encouraging preclinical studies have led to the initiation of various translational projects to bring gene supplementation therapies for *CNGA3*- and *CNGB3*-linked ACHM to the clinics (Table 1). The first study to recruit patients was a phase I/II exploratory, dose-escalation study, which tests safety and efficacy of a single dose subretinal injection of rAAV8.hCNGA3 in patients with ACHM caused by *CNGA3* mutations (clinicaltrials.gov identifier: NCT02610582) [118]. This clinical trial was conducted by the RD-CURE consortium encompassing clinicians and researchers from the University Eye Hospital Tübingen and the Ludwig-Maximilians-University Munich. Nine patients were treated in this trial and publication of study results is expected in 2018. Three additional interventional phase I/II clinical trials with ACHM patients are currently in progress. Two studies concern *CNGB3*-linked ACHM (NCT02599922 and NCT03001310) and are sponsored by Applied Genetic Technologies Corp (AGTC) and MeiraGTx UK II Ltd., respectively. These studies also have a non-randomized, open-label and dose-escalation design and test safety and efficacy of rAAV2tYF-PR1.7-hCNGB3 (AGTC study) or AAV2/8-hCARp.hCNGB3 (MeiraGTx study) in patients with ACHM caused by mutations in *CNGB3*. Both studies started enrolling patients in 2016. Another phase I/II clinical trial testing safety and efficacy of rAAV2tYF-PR1.7-hCNGA3 in patients with *CNGA3*-linked ACHM sponsored by AGTC (NCT02935517) enrolled the first patient in May 2017. These studies together bring hope to patients with CNG channel-linked eye diseases and suggest that gene therapy products targeting CNG channelopathies could follow the path of Luxturna^®^ (voretigene neparvovec-rzyl), the first in class rAAV gene therapy product for the treatment of biallelic *RPE65* mutation-linked retinal dystrophy, which recently received marketing authorization from the U.S. Food and Drug Administration. Pricing negotiations for Luxturna^®^ are ongoing and we will have to wait and see how affordable such treatments will be. 

## Figures and Tables

**Table 1:** Overview of retinal CNG genes, associated human diseases, animal models, preclinical and clinical gene therapy studies. ID, www.clinicaltrials.gov identifier. POC, proof-of-concept.

Gene (Cell Type)	Associated Human Disease	Animal Models	POC Studies	Preclinical Safety Studies	Clinical Trials (ID)
*CNGA1* (rods)	Retinitis pigmentosa, RP49	*Cnga1* antisense expressing mice: retinal degeneration [53] Canine model (missense mutation): Progressive retinal atrophy [54]	-	-	-
*CNGB1* (rods)	Retinitis pigmentosa, RP45	*Cngb1*-deficient mice: impaired rod function and retinal degeneration [55,56] Canine model (missense mutation): impaired rod function and retinal degeneration [89]	[58,90]	-	-
*CNGA3* (cones)	Achromatopsia, ACHM2	*Cnga3*-deficient mice: loss of cone function and cone degeneration [74] *cpfl5* mouse: loss of cone function and cone cell degeneration [91] Sheep model (missense mutation): loss of cone function and day blindness [80]	[76,78,92,93,94,95,96]	[97,98,99,100]	NCT02610582 NCT02935517
*CNGB3* (cones)	Achromatopsia, ACHM1	Canine models (null-deletion or missense mutation): cone degeneration [84] *Cngb3*-deficient mice: Impaired cone function and cone degeneration [82]	[101,102,103]	[98,104,105,106]	NCT02599922 NCT03001310
